# The effects of delayed annealing on the luminescent activity of heavy metal cadmium zinc phosphate glasses activated by: Er^3+^ and Tb^3+^ ions

**DOI:** 10.1038/s41598-024-55409-7

**Published:** 2024-03-01

**Authors:** M. A. Marzouk, F. H. Elbatal, H. A. Elbatal, I. M. Elkashef

**Affiliations:** 1 Glass Research Department, National Research Centre, 33 El Bohouth Street (former EL Tahrir), Dokki, P.O. 12622, Giza, Egypt; 2 Physics Department, Faculty of Science, Arish University, Arish, Egypt

**Keywords:** Phosphate, Heavy metal oxide, Glass, Rare-Earth, Annealing, Luminescence, Glasses, Optical manipulation and tweezers

## Abstract

The luminescent spectra of the RE_2_O_3_-doped P_2_O_5_–CdO–ZnO glasses (RE = Er, and Tb) were investigated to separate the effects of two studied rare-earth elements and the annealing regime on the emission performance of the prepared glasses. The glasses undergo a series of collective measurements including UV–visible absorption, luminescence, thermal expansion, XRD, TEM, and FTIR. The optical UV–visible spectra of the two doped glasses reveal a UV band due to undoped glass beside and extra extended 11 peaks with the Er^3+^ ions with high distinct features while the Tb^3+^ ions samples exhibit peaks within the visible region. These peaks are correlated with transitions from the ground state in each case to specific energy transitions. The overall optical data indicate that the two rare earth ions are present in a stable trivalent state. Under UV excitation, both Er^3+^ and Tb^3+^ emit a characteristic green light corresponding to ^4^S_3/2_ → ^4^I_15/2_ and ^5^D_4_ → ^7^F_5_ transitions, respectively. The performance of the green light was identified to be enhanced by increasing the concentration of rare earth and the effect of annealing temperature. Moreover, the intensity of the infrared emission of Er^3+^ at 1532 nm corresponds to the (^4^I_13/2_ → ^4^I_15/2_) transition which is assumed to be developed with the effect of heating. The resultant IR spectra show distinct vibrational peaks due to phosphate groups that undergo only minor modifications when doped with rare earth elements or over-annealed.

## Introduction

Phosphate glasses are grouped as one of the most distinctive and extensive studies of inorganic vitreous candidates beside silicates and borates^1^. Phosphate glasses resemble silicate glasses by retaining the tetrahedral structural units (PO_4_) and (SiO_4_) within the entire composition limits while borate glasses exhibit trigonal BO_3_ and BO_4_ groups^1–4^. The chemical durability of phosphate glasses is identified to be improved by the introduction of multivalent oxides to their composition as evidenced by the high efficiency of lead iron phosphate glasses more than traditional borosilicate glasses as evidenced by some researchers^4,5^.

Some scientists^5–7^ have assumed that alkali phosphate glasses possess reducing power initiating the presence of some transition metal ions in the low valence or octahedral coordination in reverse to alkali silicate and alkali borate glasses which promotes the tetrahedral coordination or high valence.

The introduction of either rare earth ions or 3d transition metal ions to glass produces valuable optical properties expressed by the characteristic absorption bands at specific wavelengths which can be utilized in various optical applications. It is evident that host glasses are the best media for rare earth ions in optical materials due to the easiness of preparation including shaping and reaching optical homogeneity, lower non-linear refractive index, and can dissolve measurable concentrations of RE^3+^ ions^8–10^.

It is recognized that rare-earth (lanthanides) are separated into two groups; namely, the cerium group (Ce–Gd) and the terbium group (Tb–Lu)^11,12^. The first group (cerium group) is called “lighter lanthanides” and the second group (terbium group) is called “heavier lanthanides”.

Generally, the annealing process of glass melts is a regulated cooling process that helps to release the internal stress from the final glass product. This procedure is crucial to glass production because it improves the chemical, mechanical, and thermal qualities of glass. Moreover, the superior annealing process of glass enhances its optical properties. Briefly, annealing is considered an essential step that makes the glass stronger and more durable. In the current study, we aimed to study the photoluminescence strength of the green emission from Eu^3+^ or Tb^3+^ under normal and different annealing temperatures with a sufficient fast cooling rate to form the amorphous phase and avoid the crystallization of a melt.

As previously noted, the presence of rare earth elements and thermal annealing both have an impact on the luminous qualities of glass. An appropriate glass annealing process offers an essential way to investigate the reduction of intrinsic flaws in an amorphous state and the amorphous-to-crystalline transition. This might aid in the development of RE-containing glasses for luminescent components like light-emitting diodes and optical devices. Research into enhancing the optical behavior of RE-doped phosphate glasses is prompted by the aforementioned properties. Hence, the main objective of the current work is to make a characterization and evaluate the efficiency of the luminescence behavior of the prepared heavy metal cadmium zinc phosphate glass activated by one of the two common rare earth ions Er^3+^ and Tb^3+^ after a controlled annealing process without reaching the crystalline nature of the prepared glasses. Hence, in our present work, we focused our study on the two elements Tb^3+^ and Er^3+^. The present work comprises the preparation of rare earth doped (Er^3+^, Tb^3+^) with two ratios (0.5 and 1%) in host phosphate glasses of the composition 70P_2_O_5_–15CdO–15ZnO in mol% and including the measurements of their optical, FTIR, luminescence properties to evaluate the state of the two rare earth ions and their behavior in this mentioned specific phosphate glass system.

## Experimental details

### Glass preparation

The glasses of the batch composition listed in Table 1 were prepared from laboratory chemicals including (NH_4_H_2_PO_4_) for P_2_O_5_, CdCO_3_ for CdO, and ZnO as such. The applied melting temperature was at 1200 °C for 2 h including frequent rotating the melts to reach complete mixing and homogeneity. The melted glasses were poured into a warmed stainless-steel mold and immediately transferred to annealing a muffle furnace.

**Table 1:** Chemical composition in mol% of the studied glasses.

Sample	Composition in mol%					E_opt_ (eV)
	P_2_O_5_	CdO	ZnO	Er_2_O_3_	Tb_2_O_3_	
1	70	15	15	–	–	4.921
2	70	15	15	0.5	–	4.240
3	70	15	15	1	–	3.674
4	70	15	15	–	0.5	3.250
5	70	15	15	–	1	3.162

Regarding the annealing process, the first group of samples was cast and placed in an annealing muffle furnace at a temperature of 320 °C. The muffle furnace was then switched off immediately after placing the samples and left until it reached room temperature. Another group of the same samples was also poured and placed directly in an annealing muffle furnace at a temperature of 650 °C. The muffle furnace was then switched off immediately after placing the samples at an average cooling time of 4–6 h for the two groups.

A muffle furnace adjusted at 320 °C. The annealing muffle was switched off after 1 h and left to cool with the samples inside. Another set of glass samples has been annealed at different two stepwise temperatures approximately equal to 320 °C then 650 °C for a total holding time 6 h.

### Characterizations

FTIR absorption spectra were measured by a spectrometer type (FTIR Brucker Vertex 8V, Germany) using the KBr disc technique.

The thermal expansion data of the glassy samples were measured using a computerized dilatometer type (NETSCH-402 PC, Germany) with a heating rate of 5 °C/min. The thermal expansion measurements were carried out from room temperature up to the dilatometric softening temperature of each glass.

X-ray diffraction analysis was performed by a diffractometer (type Philips PW 1390) using a Ni-filter and a Cu-target was used to analyze powdered samples.

Scanning electron microscopy (SEM) was used to determine the microstructure of the produced phases. The Quanta Field Emission Gun (FEG) 250 (FEI company) high-resolution scanning electron microscope (HSEM) instrument used an accelerating voltage of 30 K.V., magnification ranging from 10× to 400,000×, and resolution for wavelength (3.5 nm) to detect the elemental analysis.

The UV–visible absorption spectra of the studied glasses were conducted using a recording spectrophotometer (type Jasco 570, Japan) within the range 200–1100 nm for polished samples with equal thickness (2 mm ± 0.1 mm).

A fluorophotometer (type JASCO, FP-6500, Japan) outfitted with a xenon arc lamp as the excitation light source was used to measure the emission and excitation of the prepared glasses at room temperature and under various excitation wavelengths in the spectral range 200–750 nm.

The created glasses’ and their related glass–ceramic samples' emitted light colors were estimated using color coordinates. The CIE1931 color chromaticity diagram shows the colors that light emits by using the three primary dimensionless quantities x, y, and z to calculate the tristimulus values as shown below^13,14^:

1 $${\text{X}} = { }\int {\text{P}}\left( {\uplambda } \right){\overline{\text{x}}}\left( {\uplambda } \right){{\text d}{\uplambda }}$$

2 $${\text{Y}} = { }\int {\text{P}}\left( {\uplambda } \right){\overline{\text{y}}}\left( {\uplambda } \right){{\text d}{\uplambda }}$$

3 $${\text{Z}} = { }\int {\text{P}}\left( {\uplambda } \right){\overline{\text{z}}}\left( {\uplambda } \right){{\text d}{\uplambda }}$$

The following formula can be used to estimate the color chromaticity coordinates x and y, where P(λ) is the spectral relative power and X, Y, and Z are tristimulus values^13,14^;

4 $${\text{x }} = { }\frac{{\text{X}}}{{{\text{X}} + {\text{Y}} + {\text{Z}}}}$$

5 $${\text{y }} = { }\frac{{\text{Y}}}{{{\text{X}} + {\text{Y}} + {\text{Z}}}}{ }$$

6 $${\text{z }} = { }\frac{{\text{Z}}}{{{\text{X}} + {\text{Y}} + {\text{Z}}}}{ }$$

## Results and discussions

### Optical absorption spectra

**Figure 1:**
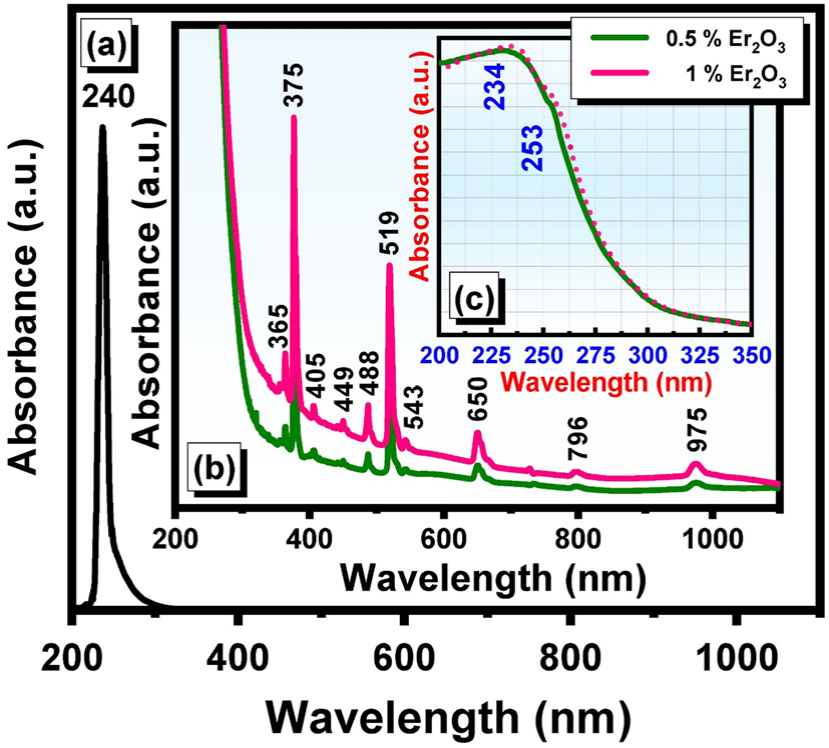
UV–visible absorption spectra of undoped and Er_2_O_3_ doped cadmium zinc phosphate glasses where (**a**) undoped, (**b**) UV–visible full absorption range, and (**c**) magnification of 200–300 range.

The absorption spectra of the undoped glass (Fig. 1a) reveal one characteristic UV absorption band centered at about 240 nm without any extended absorption bands in the visible region. This UV absorption maximum at 240 nm is assumed to be caused due to remnants of impurities from transition metal ions (such as Fe^3+^ and Cr^6+^) that were contaminated within the chemicals used to prepare the glasses, and numerous glass scientists now concur with these earlier postulations^15–17^.

Figure 1b illustrates the UV–visible spectra of the undoped and two Er_2_O_3_—doped glasses (0.5 and 1 mol%). The detailed absorption spectra identified are extended from 365 to 975 nm after the UV peaks at 234 and 253 nm from the base undoped sample as shown in the inside Fig. 1c. The peaks are observed at 365, 375, 405, 449, 488, 519, 543, 650, 729, 796 and 975 nm. The three peaks are evidently higher in intensity at 375, 488, and 519 nm. It is also observed that the increase in the Er_2_O_3_ content reveals an obvious increase in the intensity of all the identified peaks.

The optical properties of Er^3+^ ions have been studied in various glasses and in crystalline hosts^18,19^. The absorption spectra exhibit the typical rare–earth ion spectra involving an array of bands extending from the UV to the visible regions. The origin of absorption spectra of rare-earth ions is very similar and close to a measurable degree due to the fact their outermost electronic structure is the same. The different elements differ, however in the electron population in their inner 4F shells and indeed is formed by the progressive electronic filling up to that shell. The majority of the rare-earth ions when introduced into glass, introduce very sharp and distinctive absorption bands, especially in the visible-near IR region. The identified specific spectra are due to electronic transitions between energy levels in the 4F shell. Their sharpness is due to the fact that the inner electronic shell is largely shielded from the effects of the ligand field by the outer 5s and 5p electrons. The absorption spectra of Er_2_O_3_ -doped glasses and their transitions are listed in Table 2. It is very important to mention that the identified distinct optical absorption peak transitions in this work are very close and similar to those observed in various host PbO–ZnO–CdO glass^18–20^. This supports the view that rare–earth ions are shielded and the trivalent Er^3+^ ions are stable and not reduced to lower valence.

**Table 2:** Depicts the identified UV–visible absorption peaks of the studied host P_2_O_5_–ZnO–CdO glass system co-doped with Er^3+^ and Tb^3+^.

Er^3+^—doped glass		Tb^3+^—doped glass	
Peak position (nm)	Energy transition ^4^I_15/2_ →	Peak position (nm)	Energy transition ^7^F_6_ →
253	^2^G_7/2_	314	(^5^G_2_ + ^5^L_6_)
365	^4^G_9/2_	326	(^5^G_5_ + ^5^L_9_)
375	^4^G_11/2_	348	(^5^G_5_ + ^5^D_2_)
405	^2^G_9/2_	371	^5^L_10_
449	^4^F_5/2_	395	(^5^D_3_ + G_6_)
488	^4^F_7/2_	483	^5^D_4_
519	^2^H_11/2_		
543	^4^S_3/2_		
650	^4^F_9/2_		
796	^4^I_9/2_		
975	^4^I_11/2_		

**Figure 2:**
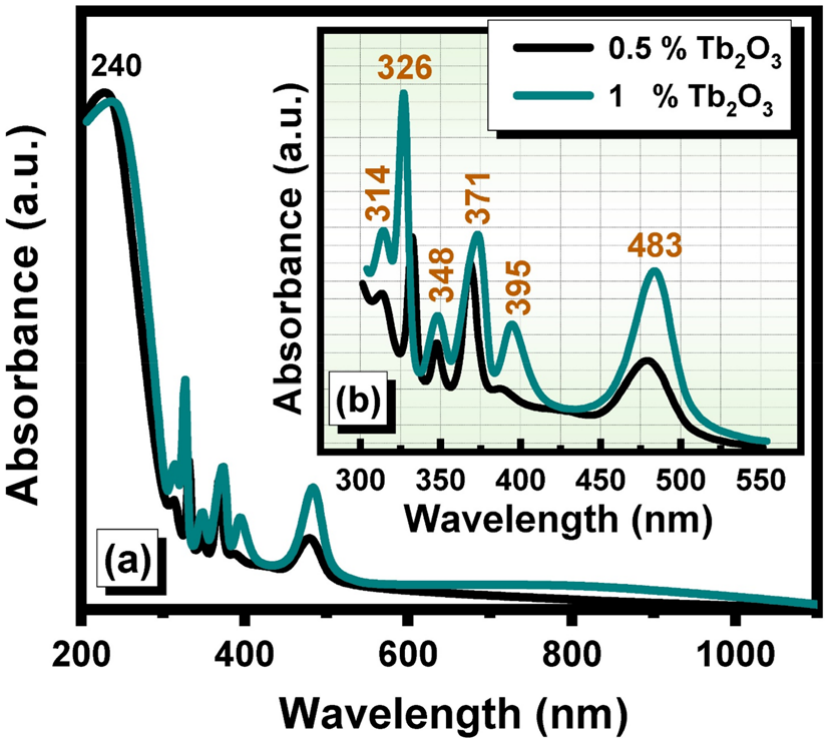
UV–visible absorption spectra of undoped and Tb_2_O_3_ doped cadmium zinc phosphate glasses where (**a**) UV–visible full absorption range and (**b**) Magnification of 300–550 range.

Figure 2 shows the optical spectra of the glasses with Tb_2_O_3_ (0.5, and 1 mol %). The identified spectra are not extended as that for Er_2_O_3_ glasses but reveal the following peaks with the beginning in the peak at 240 nm due to base undoped followed by peaks at 314, 326, 348, 371,395, and 485 nm. The high-intense peaks are identified at 326, 391, and 483 nm. Interpretations of the optical spectra of the Tb_2_O_3_–doped glasses. Previous studies based on Tb^3+^—doped glasses^20–23^ arrived to the attributions as listed in Table 2 derived from optical bands from the ^7^F_6_ ground state.

The optical bandgap values were estimated according to Tauc’s plot. The method of estimation with details is given in reference^24^. Table 1 presents the estimated optical bandgap of the prepared glasses. The recorded optical bandgap values exhibit a characteristic decrease that corresponds to the content of RE in the batch composition of the samples. The E_opt_ values were noted, at 4.921 eV for the undoped samples while it decreases to its minimum value with the addition of Er_2_O_3_ or Tb_2_O_3_. The majority of the results were in close agreement with previously reported RE^3+^ ion-doped phosphate glasses^24^.

### Luminescent characteristics

#### Er_2_O_3_–doped glasses

**Figure 3:**
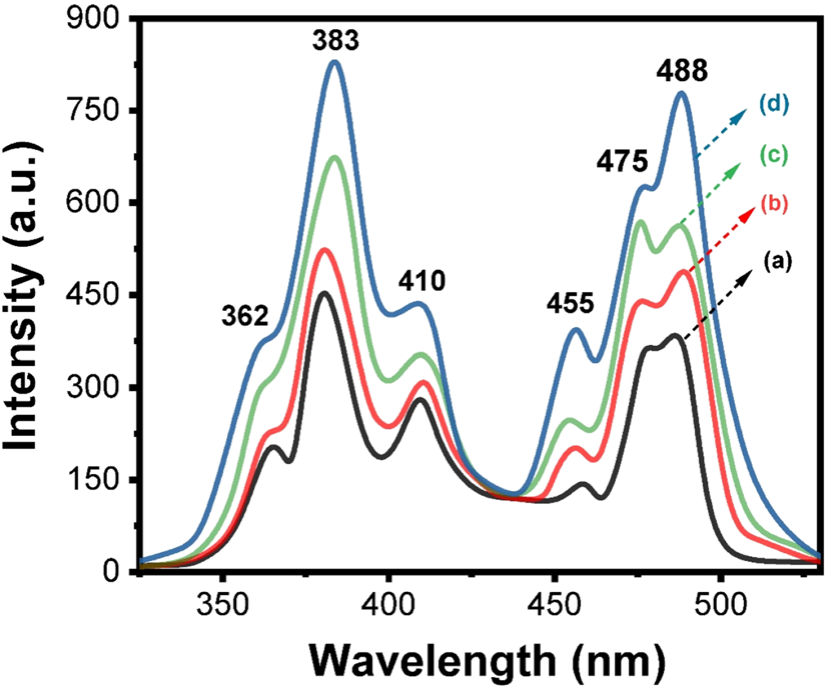
Excitation spectra of Er_2_O_3_ doped glasses where (**a**) 0.5 mol % and (**b**) 1 mol % annealed at 320 °C while (**c**) 0.5 mol % and (**d**) 1 mol % annealed at 650 °C.

The excitation spectra of 0.5 and 1% Er_2_O_3_–doped cadmium zinc phosphate glasses annealed at 320 °C together with the glassy sample annealed at 650 °C are represented in Fig. 3. According to the excitation measurements at 545 nm, the spectra reveal five different excitation bands located at 362, 383, 410,455, and 475–488 nm and correspond to the transitions of ^4^I_15/2_ → ^4^G_9/2_, ^4^G_11/2_, ^2^G_9/2_, ^4^F_5/2_ and ^4^F_7/2_ respectively^24,25^. Considering the intensity of the excitation bands, the band at 383 nm, which is substantially more intense than the other transitions among them, is employed to stimulate the Er^3+^ doped cadmium zinc phosphate glasses.

**Figure 4:**
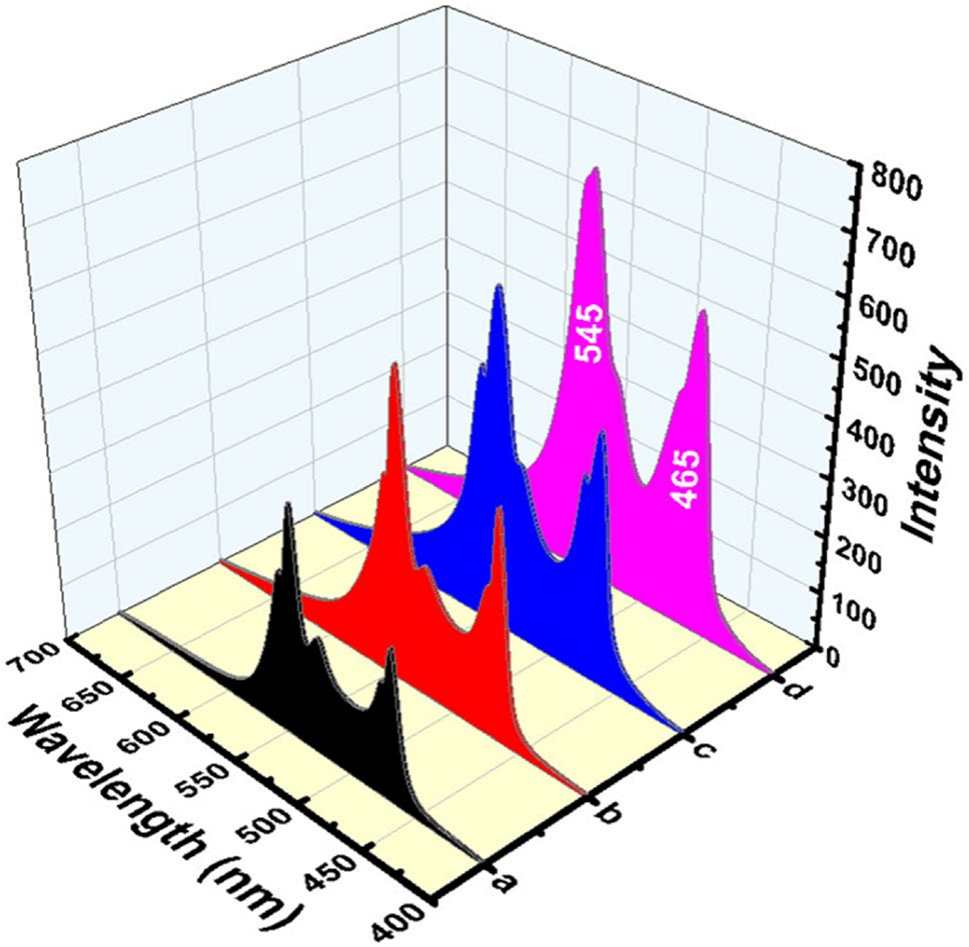
Emission spectra of Er_2_O_3_ doped glasses where (**a**) 0.5 mol % and (**b**) 1 mol % annealed at 320 °C while (**c**) 0.5 mol % and (**d**) 1 mol % annealed at 650 °C.

Figure 4 shows the emission spectra of the prepared Er^3+^-doped glass with an excitation wavelength of 383 nm from which collective strong emission bands in the visible region have been detected. The spectra consist of strong emissions at 465–475, 521, and 545 nm corresponding to ^4^F_5/2_ → ^4^I_15/2_, ^2^H_11/2_ → ^4^I_15/2_, and ^4^S_3/2_ → ^4^I_15/2_ transitions respectively^24–26^. The results show a characteristic progressive increment in the intensity of excitation and emission spectra due to the effect of the Er_2_O_3_ content and the annealing temperature.

**Figure 5:**
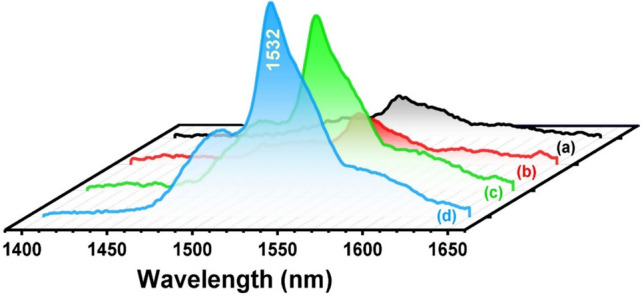
NIR-emission spectra at 980 nm laser excitation of Er_2_O_3_ doped glasses where (**a**) 0.5 mol % and (**b**) 1 mol % annealed at 320 °C while (**c**) 0.5 mol % and (**d**) 1 mol % annealed at 650 °C.

The near-infrared fluorescence spectra of Er^3+^ -doped glasses upon laser excitation at 980 nm are shown in Fig. 5. From these spectra, a characteristic broad near-infrared emission centered at 1532 nm corresponds to (^4^I_13/2_ → ^4^I_15/2_) transition. The intensity of the emission band is increased with the concentration of Er_2_O_3_ and with thermal treatment. The obtained NIR-emission (1.5 μm) from Er^3+^ -doped glasses is correlated to the ^4^I_11/2_ → ^4^I_15/2_ state, which in turn nonradiative relaxes to ^4^I_13/2_ Stark-split levels to the ground level at applying the laser excitation 980 nm^24,26,27^.

#### Tb_2_O_3_—doped glasses

**Figure 6:**
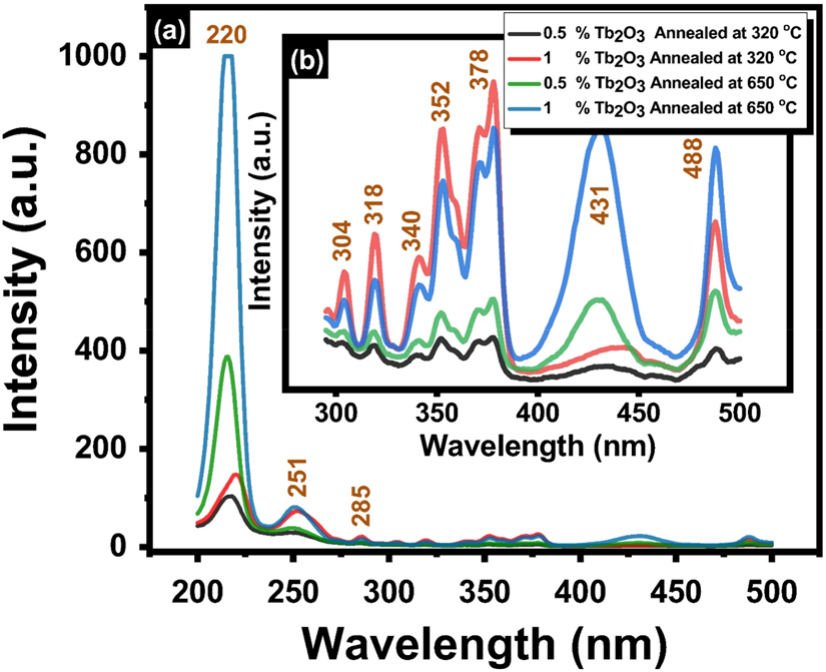
Excitation spectra of 0.5 and 1% Tb_2_O_3_ doped glasses at different annealing temperatures where (**a**) covers the total range 200–500 nm and (**b**) magnification for spectrum range 300–500 nm.

The excitation spectra of the prepared Tb_2_O_3_-doped cadmium zinc phosphate glasses are shown in Fig. 6. The results of measurements indicated that the maximum excitation peak is located at 220 nm, with other extended peaks located at 250, 285, 304, 318, 340, 352, 378, 431, and 488 nm. The strongest band centered at 220 nm corresponds to the transitions from the lower ground level ^7^F_6_ of the 4f^8^ configuration to the higher spin–allowed 4f^7^ 5d1 level of the Tb^3+^ ion^28,29^, while the other extended excitation peaks are associated with transitions from the ^7^F_6_ ground state to (^5^I_8_, ^5^F_4_, ^5^F_5_, ^5^H_4_), (^5^H_5_, ^5^H_6_), (^5^H_7_, ^5^D_1_), (^5^L_7,8_, ^5^G_3_), (^5^L_9_, ^5^D_2_, ^5^G_5_) 352, (^5^L_10_, ^5^G_6_, ^5^D_3_), ^5^D_4_ for 284,304,318, 340,377, and 488 respectively^28–33^. The attribution of the band centered at 431 nm was related to the absorption of the glass network matrix^33^. For the measurement of emission spectra, the significant strong excitation peak near 220 nm (^7^F_6_ → 4f^7^ 5d1) has been selected.

**Figure 7:**
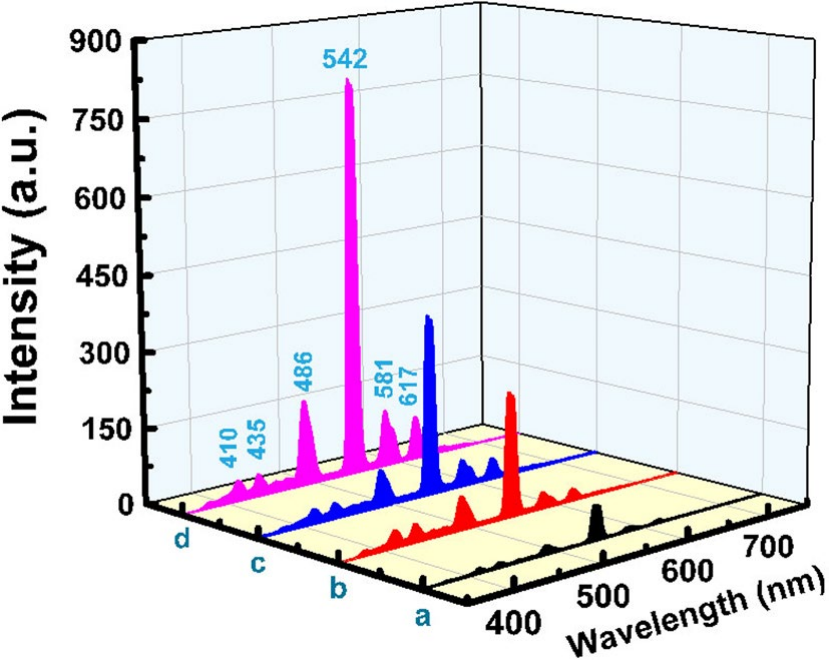
Emission spectra of Tb_2_O_3_ doped glasses where (**a**) 0.5 mol % and (**b**) 1 mol % annealed at 320 °C while (**c**) 0.5 mol % and (**d**) 1 mol % annealed at 650 °C.

The emission spectra of samples excited at 220 nm are shown in Fig. 7. They consist of six emission peaks associated with 4f^8^ → 4f^8^ transitions from the ^5^D_3_ (blue emissions) and ^5^D_4_ (green emissions) to ^7^F_J_ multiple levels of Tb^3+^. Specifically, for the present study, the emission peaks centered at 410, 435, 486, 542, 581, and 617 nm correspond ^5^D_3_ → ^7^F_5_, ^5^D_3_ → ^7^F_4_, ^5^D_4_ → ^7^F_6_, ^5^D_4_ → ^7^F_5_, ^5^D_4_ → ^7^F_4_ and ^5^D_4_ → ^7^F_3_ transitions respectively^28–33^. The most intense emission is concentrated at 542 nm due to ^5^D_4_ → ^7^F_5_ transition which is characteristic of prominent green emission. Regarding the photoluminescence spectra of Er_2_O_3_ or Tb_2_O_3_, the intensity of the peaks increased with the increase of the content of Tb^3+^ or Er^3+^ in emission spectra, and no concentration quenching of both rare -earth is observed, in addition, the thermally annealed samples at 650 °C produce much stronger emission than that in the annealed glasses at 320 °C.

#### CIE chromaticity co-ordinates of Er_2_O_3_ and Tb_2_O_3_—doped glasses

**Figure 8:**
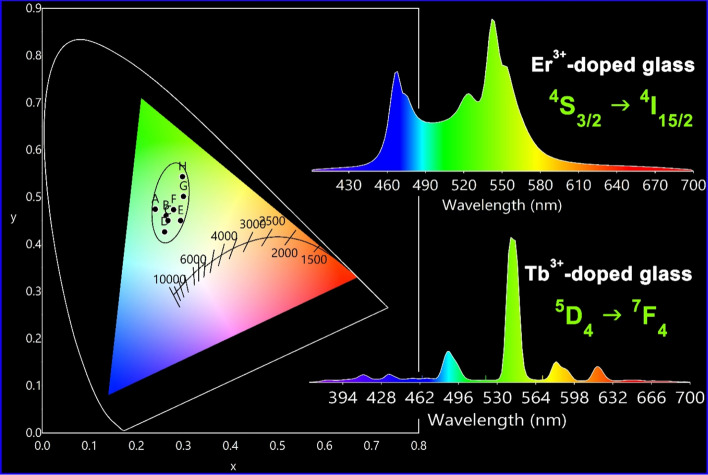
CIE-chromaticity diagram and overview emission spectra of Er^3+^ and Tb^3+^—doped glasses (details of coordinates are listed in Table 3).

The CIE color of the prepared cadmium zinc phosphate glasses closed to the green area (elapsed shape) by increasing the Er^3+^ or Tb^3+^ content and the effect of heating as can be appreciated from Fig. 8. Table 3 lists the specific CIE color coordinate values for each sample located in Fig. 8. Er_2_O_3_ doped glasses have a strong green lighting color with hue blue lighting which may interpret the tendency to the lower part of the elapsed green area. On the other hand, Tb_2_O_3_–doped samples tend to appear in the highest part of the elapse. The emissions are located at about 545 nm for both glasses and the intensity is more intensive with increasing the rare-earth content and enhanced after annealing the samples at higher temperature. The results indicated the ability of the prepared glasses to be applicable in the field of solid-state green laser.

**Table 3:** CIE chromaticity coordinates of glass samples annealed at 320 °C and 650 °C (the codes match as shown in Fig. 8).

Sample	Code	CIE coordinates	
		x	y
0.5% Er_2_O_3_ glass at 320 °C	A	0.240	0.474
1% Er_2_O_3_ glass at 320 °C	B	0.263	0.460
0.5% Er_2_O_3_ glass at 650 °C	C	0.267	0.450
1% Er_2_O_3_ glass at 650 °C	D	0.260	0.426
0.5% Tb_2_O_3_ glass at 320 °C	E	0.294	0.450
1% Tb_2_O_3_ glass at 320 °C	F	0.279	0.473
0.5% Tb_2_O_3_ glass at 650 °C	G	0.300	0.501
0.5%Tb_2_O_3_ glass at 650 °C	H	0.298	0.543

### Thermal expansion characteristics

**Figure 9:**
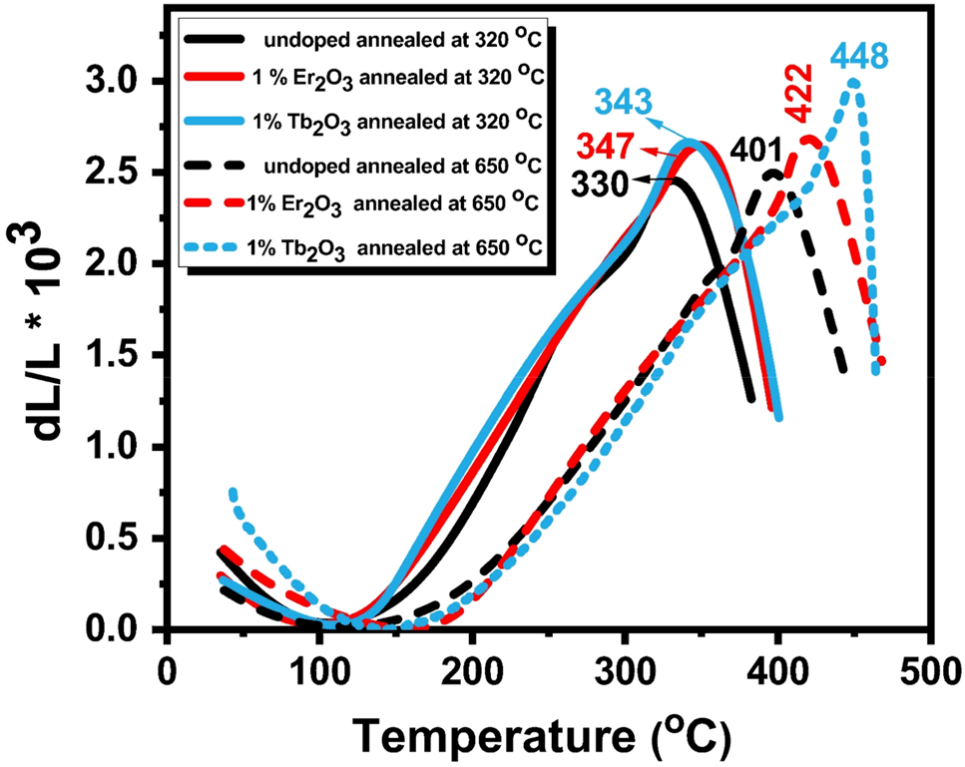
Thermal expansion of the selected 1% rare–earth doped glass at 320 and 650 °C annealing temperatures.

The values of the coefficient of thermal expansion (CTE), glass transition temperature (T_g_), and dilatometric softening temperature T_d_ were obtained from the thermal expansion curves (Fig. 9) and the data are collected in Table 4. The undoped glass sample reveals thermal parameters equal to 7.6 × 10^–6^ (/°C), 302 ℃, and 330 ℃ for CTE, T_g_, and T_d_ respectively. When 1% of the rare earth is introduced into the glass otherwise Er_2_O_3_ or Tb_2_O_3_, the detected thermal parameters (T_g_ and T_d_) were increased than that of the undoped glasses. As evident with the progressive annealing process of the samples, the previous parameters are simultaneously increased.

**Table 4:** Thermal parameters of undoped and 1 mol% rare earth-doped zinc cadmium phosphate glasses annealed at 320 °C and 650 °C.

Sample code	T_g_ (°C)	T_d_ (°C)	CTE (10^–6^/°C)
Undoped glass at 320 °C	302	330	7.60
1% Er_2_O_3_ glass at 320 °C	319	347	7.09
1% Tb_2_O_3_ glass at 320 °C	311	343	4.60
Undoped glass at 650 °C	373	401	5.61
1% Er_2_O_3_ glass at 650 °C	392	422	2.40
1% Tb_2_O_3_ glass at 650 °C	412	440	3.40

When modifiers or other compounds are added, the immiscibility temperature is concurrently suppressed and the glass transformation temperature is raised. This results in the elimination of metastable immiscibility at all temperatures where slow kinetics are not inhibiting it^3^. The increase in thermal expansion coefficient arises from the creation of a non-bridging oxygen from a bridging oxygen that increases the asymmetry of the bond to the neighboring forming cations, or other network cations. Bonds between an anion and two neighboring cations of different field strength are less symmetric than bonds involving neighboring cations of equal field strength^3^.

According to Saetova et al.^34^ CTE of glasses depends on the degree of network polymerization and its strength. Since rare-earth oxides are assumed to act as modifiers. The increment in thermal parameters with a concentration of the rare–earth oxide can be attributed to the progressive formation of non-bridging oxygens (NBOs) which should exhibit a higher value of CTE^35–37^. Regular expansion is defined as the reaction to the constituents’ amplitude-increased atomic vibrations during heating. Hence significant shifts in the dilatometric softening temperature to higher values after thermal treatment may have been brought on by a reduction in the stress that is generated in glass during quenching or cooling during synthesis^36,37^.

### XRD and morphological characteristics

**Figure 10:**
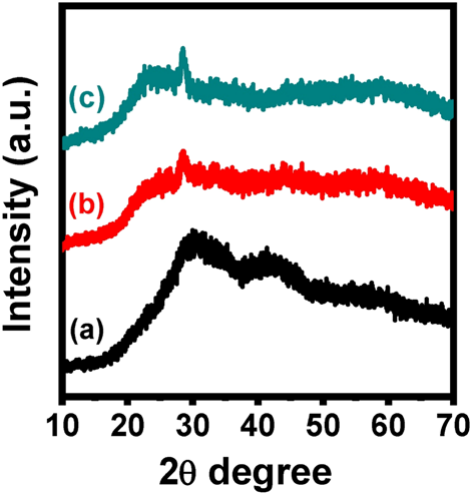
XRD of the thermally heated glasses where (**a**) undoped, (**b**) 1% Er_2_O_3_, and (**c**) 1% Tb_2_O_3_—doped glasses after annealing at 650 °C.

Figure 10 shows the XRD patterns of annealed samples at 650 °C, which do not exhibit sharply diffracted separated peaks but instead display broad humps, indicating the amorphous or non-periodic nature of the samples. Although this is common and illustrates the structural disorder in the samples, broad diffusion is seen in the samples at lower scattering angles. The amorphous behavior of the produced glass samples is revealed by the XRD measurements.

**Figure 11:**
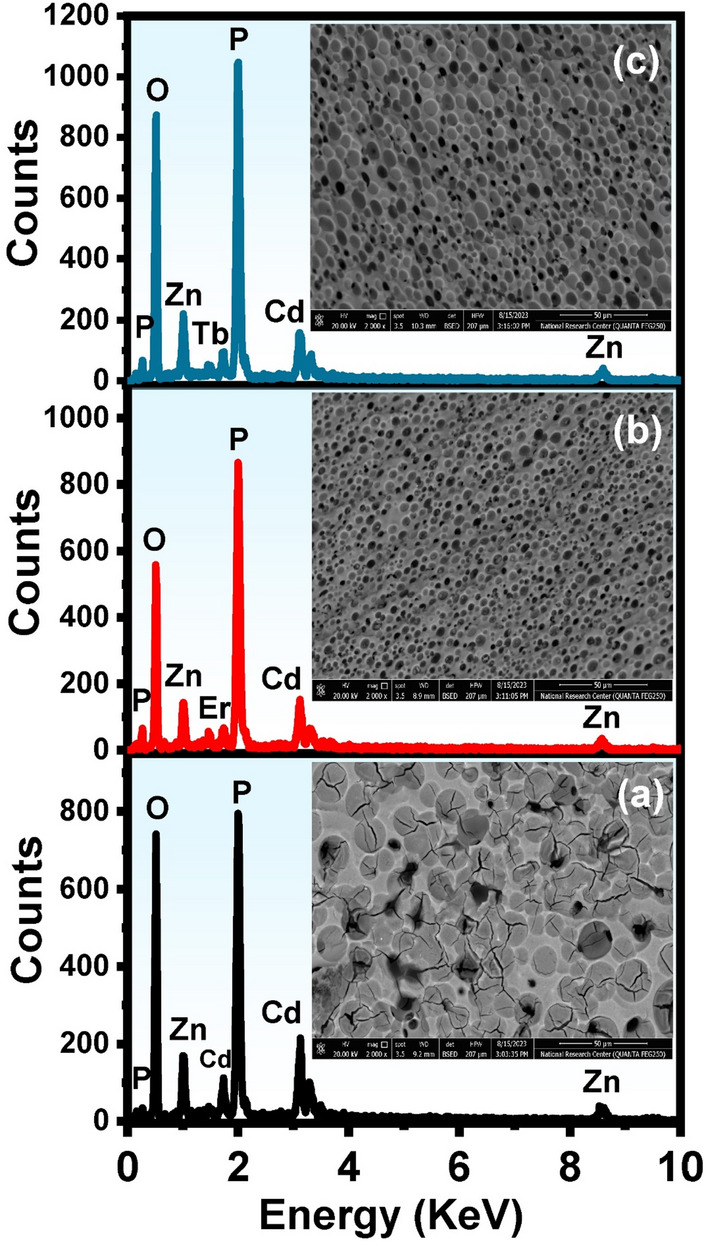
SEM and EDAX spectrum of the thermally treated glasses where (**a**) undoped, (**b**) 1% Er_2_O_3_, and (**c**) 1% Tb_2_O_3_—doped glasses after annealing at 650 °C.

High-resolution scanning electron microscopy (HRSEM) images were taken to examine the surface characteristics and EDX spectra were taken for elemental analysis of produced samples as shown in Fig. 11. In general, it was noted that when SEM images in Fig. 11a are investigated; it is observed that the glass surfaces are not homogenous and there are large and small structural forms like holes or circles that are not located in the structure. The morphological feature of round holes in the samples could be attributed to cluster aggregation after enhanced annealing treatment at a higher temperature. This implies that raising the treatment temperature causes the glass material's concentration to rise gradually, which in turn causes the cluster size to increase. in good accordance with the results of XRD.

The addition of rare earth causes a regular uniform distribution of zinc cadmium phosphate particles the porosity or lacunar areas are relatively regular as observed in Fig. 11b and c. On the other hand, it has been understood from EDAX spectral analysis in Fig. 11b, that the profile of the peaks reflects the elemental composition of the glasses where oxygen, phosphorus, cadmium, and zinc elements were shown as the main constituents while the concentrations of Eu^3+^ and Tb^3+^ elements is very minor compared with the elemental concentrations of the parent sample. EDAX data indicate that the obtained glass is homogeneous and the applied elements are dissolved completely in glass.

### FT infrared spectra of the studied glasses

**Figure 12:**
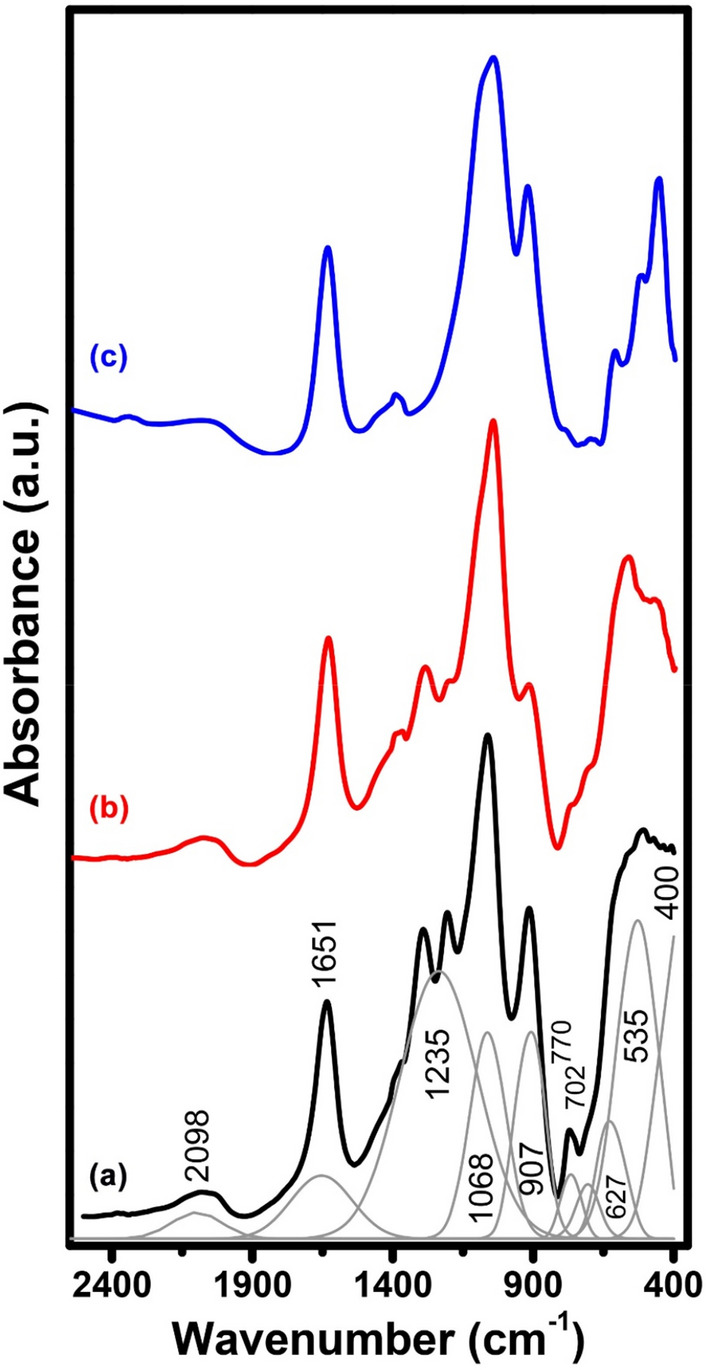
FTIR of the prepared glasses at normal annealing conditions where (**a**) undoped, (**b**) 1% Er_2_O_3,_ and (**c**) 1% Tb_2_O_3_ after annealing at 320 °C.

Figure 12 illustrates the FTIR spectra of three glasses including the base undoped sample and both Tb-doped and Er-doped glasses after annealing at 320 °C. The resultant IR absorption can be summarized as follows:

- The IR spectrum of the base undoped P_2_O_5_–ZnO–CdO glass shows extended absorption from 400 to 3000cm^−1^ with deconvoluted peaks at 400, 535, 627, 702, 770, 907, 1068, 1135, 1651, and 1698 cm^−1^.
- The addition of Tb_2_O_3_ causes some variations in the intensities of the bands at 535 and 907 cm^−1^.
- The introduction of Er_2_O_3_ brings some variations in the far IR bands observed to be split.

**Figure 13:**
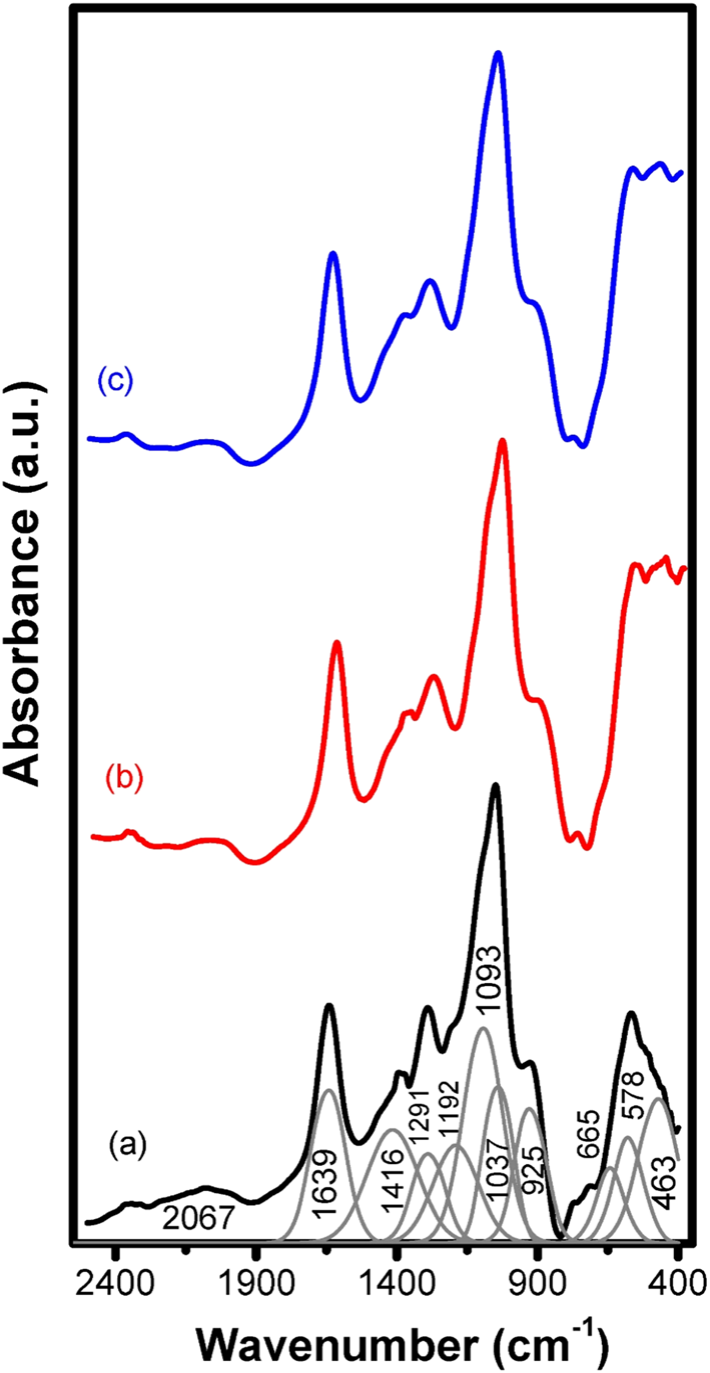
FTIR of the thermally treated 1% rare-earth doped—glasses where (**a**) undoped, (**b**) Er_2_O_3,_ and (**c**) Tb_2_O_3_ after annealing at 650 °C.

Figure 13 shows the IR spectra of the glasses annealed at 650 °C, the IR spectra extended to the same wavenumber as that of the glasses spectra from 400 to 2400 cm^−1^ and showing peaks with sharp edges. The identified deconvoluted peaks for the base also are at 463, 578, 665, 923, 1037, 1093, 1192, 1291, 1416, 1639, and 2067 cm^−1^_._

The resultant IR spectra of the studied glasses reveal prominent vibrational peaks which show limited changes with the doped rare–earth or with over-annealing. The IR data can be interpreted on the following basis^1–6^;

- It is recognized that the IR vibrational bands are due to or correlated with the structural building units in the glasses which originate or depend on the detailed chemical composition or constituents.
  The studied host base glass is composed of main P_2_O_5_ 70 mol % which refers to the presence of abundant phosphate (PO_4_) groups. The other two constituting oxides CdO and ZnO are assumed to be able to behave as conditional oxides. They are situated as modifying oxide and can form structural forming units (PO_4_) if neighboring extra oxygens are available but in the studied host base glass, they are assumed to be present in modifying states^1–6^.
- The assignments of the various identified IR peaks can be summarized as follows.
  (i) The peaks within the range 400–460 cm^−1^ can be assumed to be related to vibrations of modifying ions (Cd^2+^ and Zn^2+^).
  (ii) The peaks at 470–560 cm^−1^ can be related to the bending or harmonic vibrations of O–P–O linkages.
  (iii) The peaks at 650–7600 cm^−1^ are related to symmetric stretching vibrations of P–O–binding vibration.
  (iv) The peaks at 901–1010 cm^−1^ are assumed to originate from asymmetric stretching vibrations of P–O–P groupings.
  (v) The peaks at 1170 and 1260 cm^−1^ are assumed to be related to the symmetric stretching of P–O–P and stretching of doubly bonded P=O.
  (vi) The peaks at 1350 cm^−1^ is related to the harmonious of the P–O–P linkages.
  (vii) The peaks at 1650 cm^−1^ are due to vibrations of water, OH groups.
  (viii) The peak at 2098 cm^−1^ can be related to vibrations of water, OH, or POH.

## Conclusion

Undoped and rare-earth oxide (Er_2_O_3_ and Tb_2_O_3_)-doped glasses were synthesized within a host glass composition (70P_2_O_5_–15CdO–15ZnO) using a melting-annealing method. The annealing step includes subjecting the glass to carefully timed and controlled cooling temperatures at 320 °C and 650 °C after it has been heated to a high temperature. Undoped glass exhibited a UV absorption peak at 240 nm due to impurities from transition metal ions. Er_2_O_3_-doped glasses showed extended UV–visible-NIR absorption peaks at peaks at 253, 365, 375, 405, 449, 488, 519, 543, 650, 729, 796, and 975 nm, while Tb_2_O_3_-doped glasses exhibited specific peaks centered at peaks at 314, 326, 348, 371,395, and 485 nm. Photoluminescence spectra of Er^3+^-doped glass displayed strong emissions at 465–475 nm, 521, and 545 corresponding to ^4^F_5/2_ → ^4^I_15/2_, ^2^H_11/2_ → ^4^I_15/2_, and ^4^S_3/2_ → ^4^I_15/2_ transitions respectively, with an increased near-infrared emission at 1532 nm which corresponds to (^4^I_13/2_ → ^4^I_15/2_) transition. Tb_2_O_3_-doped samples excited at 220 nm emitted light at six wavelengths at 410, 435, 486, 542, 581, and 617 nm, indicating transitions ^5^D_3_ → ^7^F_5_, ^5^D_3_ → ^7^F_4_, ^5^D_4_ → ^7^F_6_, ^5^D_4_ → ^7^F_5_, ^5^D_4_ → ^7^F_4_ and ^5^D_4_ → ^7^F_3_ transitions respectively. Both Er^3+^ and Tb^3+^ doped glasses emitted strong green light emission the peak strengths increased with higher Tb^3+^ or Er^3+^ content and the annealing temperature. Undoped glass had thermal parameters of 7.6 × 10–6 (/°C), 302 ℃, and 330 ℃ for CTE, T_g_, and T_d_, respectively, which increased with rare-earth addition and thermal annealing. Morphological and XRD characterizations of the annealed samples at 650 °C indicated an amorphous nature with no any identified phase separation. IR spectral variations were attributed to rare-earth concentration, progressive annealing, and the effects of CdO and ZnO modifiers. In summary, the prepared RE-doped cadmium zinc phosphate glass benefits from delayed annealing to reduce internal stress, minimizing their optical distortions. This is crucial in the manufacturing of high-precision optical glass products such as lenses and other optical devices. Moreover, the higher-temperature annealed glasses exhibited substantially greater emission, suggesting potential use in solid-state green laser applications.

## Data Availability

The datasets used and/or analyzed during the current study are available from the corresponding author on reasonable request.
